# A new role for zinc in the brain

**DOI:** 10.7554/eLife.31816

**Published:** 2017-10-06

**Authors:** Brendan B McAllister, Richard H Dyck

**Affiliations:** Department of Psychology, Hotchkiss Brain InstituteUniversity of CalgaryCalgaryCanada

**Keywords:** auditory cortex, zinc, cortical gain, sound processing, principal neurons, interneurons, Mouse

## Abstract

Certain neurons in the auditory cortex release zinc to influence how the brain processes sounds.

**Related research article** Anderson CT, Kumar M, Xiong S, Tzounopoulos T. 2017. Cell-specific gain modulation by synaptically released zinc in cortical circuits of audition. *eLife* **6**:e29893. doi: 10.7554/eLife.29893

If asked to name one of the neurotransmitters that carry messages between neighboring neurons, few people would say "zinc". However, while it is common knowledge that zinc is an essential nutrient in humans and other animals (Vallee and Falchuk, 1993), it is not so well-known that certain neurons in the brain release zinc when they are activated (McAllister and Dyck, 2017). These neurons are a subset of the neurons that employ the amino acid glutamate as a neurotransmitter.

Zinc-releasing neurons and the synapses between them are found throughout a region of the brain called the neocortex that is involved in higher-order cognitive, motor and sensory activities (Brown and Dyck, 2004; Pérez-Clausell and Danscher, 1985). Although 'synaptic zinc' has often been studied in isolated cells and slices of brain tissue, it has seldom been studied in the intact brain. Now, in eLife, Charles Anderson, Thanos Tzounopoulos and co-workers at the University of Pittsburgh School of Medicine – including Manoj Kumar as joint first author with Anderson, and Shanshan Xiong, who is also at Central South University in China – report that synaptic zinc influences a process called gain modulation in the auditory cortex, which is the region of the neocortex that processes sound (Anderson et al., 2017).

Gain modulation occurs when an input to a neuron alters the relationship between the intensity of a stimulus – for example, the loudness of a sound – and the strength of the neuron’s response to the stimulus. Calcium ions flow into neurons when they are activated, so researchers often use changes in the levels of calcium ions as an indirect measure of brain activity. Anderson et al. injected mice with a virus that caused their neurons to express a protein that fluoresces in the presence of calcium ions. Placing these mice under a microscope revealed that applying a zinc-binding chemical, which intercepts the zinc released at synapses before it can act, to the brain enhanced the neuronal response to loud sounds.

To make certain that this effect was due to synaptic zinc, Anderson et al. conducted a similar experiment using mice that are unable to produce zinc transporter 3, a protein that is necessary for storing zinc in synaptic vesicles (Cole et al., 1999). The responses of auditory cortex neurons to loud sounds in these mice were enhanced compared to normal mice, indicating that synaptic zinc decreases gain across a broad, non-specific population of neurons in the auditory cortex.

By targeting the fluorescent protein to specific types of neuron, it was possible to delve deeper into the effects of zinc. Anderson et al. first examined the 'principal neurons' that form networks across brain regions. Previous work has shown that zinc generally inhibits neural responses (Vergnano et al., 2014; Kalappa et al., 2015). It was surprising, therefore, that synaptic zinc actually increases the gain of the principal neurons. Reasoning that this result might be due to zinc acting on other components of the local neural circuit that control the activity of the principal neurons, the researchers examined three types of interneuron. One of these – identifiable by its expression of the protein parvalbumin – directly inhibits the principal neurons.

The results suggest a model wherein synaptic zinc decreases the gain of the parvalbumin interneurons, which in turn disinhibits the principal neurons, thus increasing gain modulation in these neurons. This effect depends, in part, on zinc interacting with a class of glutamate receptor known as NMDA receptors. Of the four types of neuron examined, each displayed a unique pattern of gain modulation by synaptic zinc. Because these neurons interact through a complex neural circuit, Anderson et al. could not completely disentangle the specific effects of zinc on each cell type (a limitation that is inherent to the experimental methods used). Nonetheless, the study demonstrates a clear and complex role for synaptic zinc in modulating how the auditory cortex processes information.

It is not yet known whether gain modulation by zinc affects animal behavior. Synaptic zinc is not required for basic sound processing (Cole et al., 2001), but it may be important in complex tasks requiring gain modulation, such as focusing on a particular sound amidst a background of similar but irrelevant noise – essentially, the 'cocktail party problem' (Willmore et al., 2014). Additionally, synaptic zinc is present throughout the neocortex and in other brain structures such as the amygdala and hippocampus (Figure 1). Neuronal signaling by zinc could, therefore, play important roles in other brain activities far beyond the processing of sound.

**Figure 1.**
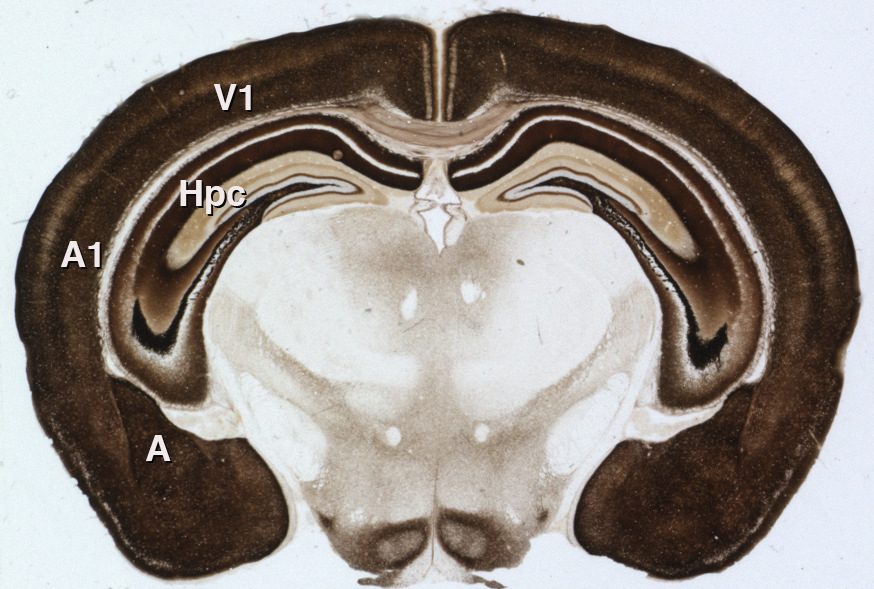
Synaptic zinc in the mouse brain. A section of mouse brain that has been stained to reveal the areas where zinc is stored at synapses (so that it can be used as a neurotransmitter). The intensity of the stain is proportional to the amount of zinc, so the darker regions contain higher levels of synaptic zinc. Hpc: hippocampus; A: amygdala; A1: auditory neocortex; V1 visual neocortex.
